# Despite shutdown hardships, remote learning may support some healthier student sleep behaviors

**DOI:** 10.1093/sleep/zsad134

**Published:** 2023-05-05

**Authors:** Benjamin L Smarr

**Affiliations:** Shu Chien Gene Lay Department of Bioengineering, University of California San Diego, La Jolla, CA, USA; Halicioğlu Data Science Institute, University of California San Diego, La Jolla, CA, USA

The COVID-19 pandemic forced colleges and universities to send students home, and to offer courses remotely. It is well established that many students are in chronobiological conflict with the schedules of courses, being required to get up earlier than desired for courses, and or staying up to finish work. The result is that college students tend on average to be sleep-deprived and socially jet-lagged [1–5], which is correlated with decreased cognitive performance [6–9] and decreased psychological well-being [8–11]. The shutdown response to COVID-19, therefore, provided a rare natural experiment in which to assess how college students would change their sleep when some of these pressures—commutes, in-person lectures, etc.—were temporarily removed and replaced with remote learning.

Rice et al., [12] analyzed sleep and light exposure data from 60 undergraduate students already generating these data as part of a class on circadian biology at the University of Washington in Seattle. Importantly, these students generated several weeks of data before, during, and after the shutdown. Perhaps most interestingly, they found that interindividual variability increased during the shutdown, but not intraindividual variability. That is, students self-selected into more diverse sleep times, without themselves becoming less stable sleepers. This is consistent with the notion that some students’ sleep timing is indeed under artificial constraints from their normal college schedules.

Consistent with this finding, the usual differences between sleep timing on weekdays and weekends went away, suggesting that students had less sleep dept and or were experiencing less weekday-imposed social jetlag when allowed more freedom in determining their sleep schedule. A final ray of light from Rice et al., [12] was that students’ light exposure was also more even across weekdays and weekends during shutdown. Given the importance of light exposure to circadian entrainment [13,14], this provides a ray of hope that students given some additional schedule control might not only sleep better, but additionally might use light to promote greater stability in their daily rhythms, including sleep but possibly affecting other positive outcomes as well. Consistent with this, Rice et al., [12] found that after shutdown, students did not completely return to pre-shutdown schedules, but instead retained an intermediate state.

The study by Rice et al., was not large—60 people cannot represent all students in all situations, even if this group was fairly diverse. And the results, while encouraging about sleep and light exposure, did not directly measure the outcomes that better sleep and daily rhythms might improve. So while the findings support the hypothesis that some version of remote learning might promote better performance and well-being, future work is needed to confirm that those effects really do follow from the observed changes.

As ambient data sources become more common, more studies like this may be possible over broader populations without substantial additional material costs. Experimenting with college schedules is not usually possible. The approach taken by Rice et al., [12] to mine existing data under interesting and relevant conditions, may provide a way around this obstacle. It is worth noting that as this study was made possible by virtue of student exercises as part of a college course on sleep and circadian rhythms. Supporting more such hands-on courses like this might lead to broader and more diverse student sleep experiences being able to be included when future natural experiments arise.
